# Role of new generation implantable loop recorders in managing undiagnosed pediatric cardiac symptoms

**DOI:** 10.1007/s00431-024-05728-8

**Published:** 2024-08-19

**Authors:** Pedro Agudo-Montore, Graham Stuart, Deirdre Wilson, Georgia Spentzou, Rabeea Sidiqqui, Cecilia González-Corcia

**Affiliations:** 1 Department of Pediatrics, Division of Cardiology, Virgen del Rocío Children Hospital, Seville, Spain; 2 Department of Pediatrics, Division of Cardiology, Bristol Royal Hospital for Children, 3175 Chem. de la Côte-Sainte-Catherine, Montréal, QC H3T 1C5, Bristol, UK

**Keywords:** Pediatric arrhythmias, Implantable loop recorder, Inherited cardiac conditions, Congenital heart disease

## Abstract

Pediatric cardiac symptoms such as palpitations, syncope, or seizure-like episodes pose diagnostic challenges for general pediatricians. These symptoms, though often benign, may reveal underlying arrhythmias or inherited cardiac conditions (ICCs), affecting the quality of life and limiting activity participation. The purpose of this study is to determine the effectiveness and safety of implantable loop recorders (ILRs) in diagnosing and managing arrhythmias in pediatric patients. A retrospective cohort study conducted over an 8-year period from January 2016 to December 2023 in a single pediatric cardiology center. A cohort of 155 pediatric patients (median age 11.4 years) who underwent ILR implantation were selected based on symptoms such as palpitations, chest pain, or syncope, and those with previously recorded arrhythmias or high-risk ICCs. The primary outcomes were the diagnostic yield of ILRs for arrhythmias and subsequent changes in patient management. Diagnostic yield was defined as the detection of relevant arrhythmias, such as pauses of 3 s or longer, high-degree AV block, sinus node dysfunction, supraventricular tachycardia, ventricular tachycardia, or inappropriate sinus tachycardia. The median follow-up period was 2.3 years (845 days). Diagnostic arrhythmias were recorded in 60% of patients with symptom-activated transmissions and 80% of device-activated transmissions. Sinus pauses (37.5%) and VT (30%) were the most common arrhythmias detected. In patients with syncope (*n* = 76), 30% had relevant arrhythmias. In the palpitations group (*n* = 20), 35% had relevant arrhythmias. Approximately 80% of patients with ILR-diagnosed arrhythmias underwent targeted management, including medication changes and additional procedures. No significant complications were observed; minor complications occurred in 2.5% of patients.

*Conclusions*: New generation ILRs are effective and safe for diagnosing and managing pediatric arrhythmias, providing significant reassurance to patients and families. Further studies are needed to evaluate the impact of ILRs on quality of life and sports participation in high-risk young patients.

**Supplementary Information:**

The online version contains supplementary material available at 10.1007/s00431-024-05728-8.

**Table:** 

**What is Known:** • *Pediatric Cardiac Symptoms: Symptoms like palpitations, syncope, and seizure-like episodes in pediatric patients pose significant diagnostic challenges.* • *Diagnostic Tools: Traditional diagnostic tools such as ECG, Holter monitors, and external loop recorders often have low diagnostic yields due to the sporadic nature of symptoms.* • *Implantable Loop Recorders (ILRs): ILRs have been shown to improve the diagnostic yield for arrhythmias in adults and provide long-term rhythm monitoring.*
**What is New:** • *Effectiveness in Pediatrics: This study demonstrates that new generation ILRs significantly enhance the diagnostic yield for arrhythmias in pediatric patients with undiagnosed cardiac symptoms.* • *Safety and Efficacy: The study confirms that ILRs are both safe and effective in the pediatric population, with minimal complications and high diagnostic accuracy.* • *Targeted Management: Findings show that ILR-diagnosed arrhythmias lead to targeted management changes in approximately 80% of cases, including medication adjustments and additional procedures.* • *Long-term Monitoring: ILRs provide continuous long-term monitoring, offering reassurance to patients and families, and potentially improving quality of life and participation in activities for high-risk young patients.*

## Introduction

In pediatric care, addressing cardiac symptoms such as palpitations, syncope, or seizure-like episodes presents unique challenges for general pediatricians and physicians. While these symptoms are typically benign in children and adolescents, they may unveil underlying arrhythmias, inherited cardiac conditions (ICCs), or exert a considerable psychological burden, limiting sport and activity participation and significantly impacting the quality of life. A comprehensive evaluation is essential to establish a diagnosis, assess risk, and formulate an appropriate management plan. Unfortunately, conventional cardiology screening methods often prove inadequate in identifying the root cause of these symptoms due to their sporadic and unpredictable nature, resulting in a low diagnostic yield with non-invasive strategies such as electrocardiogram (ECG), Holter monitoring, or external loop recorders (ELR).

The advent of implantable loop recorders (ILRs), also known as implantable cardiac monitors (ICMs), marks a significant advancement in long-term and effective monitoring for paroxysmal arrhythmias and infrequent cardiac symptoms [1]. ILRs are subcutaneous devices that provide long-term rhythm surveillance and allow documentation of the cardiac rhythm by recording electrocardiograms, both in an automatically activate modality and in a patient-activate modality [2].

An ILR system consists of three main components: the loop recorder itself, an external device to manually activate recordings during symptomatic events, and a home monitor or mobile device that transmits data to a centralized system using satellite technology. The device works by continuously monitoring heart activity and automatically records tracings when abnormal heart rhythms are detected, such as irregular, fast, or slow heart rhythms or pauses, according to predefined parameters. The settings can be customized to meet the needs and clinical characteristics of each patient, with zones set to record above the maximum normal heart rate (tachycardia zone), below the minimal heart rate (bradycardia zone), and pauses determined according to age and clinical situation [3]. Additionally, new generation ILRs can monitor and quantify the percentage (burden) of ventricular premature beats [4]. Recordings can be activated by the system when a tracing falls outside the preset “normal parameters” or manually by patients using an external device during symptomatic periods. These recordings are then transmitted wirelessly to the physician for analysis, either in person through the device programmer or remotely via a home monitor or mobile device to a web-based network.

Long-term monitoring using an ILR is recommended in highly symptomatic children when other investigations are inconclusive, due to infrequent events or the inability to complete a diagnostic protocol [5]. ILRs provide the most effective method for establishing a diagnosis when arrhythmogenic syncope is suspected but not proven despite a comprehensive evaluation [6, 7]. They are useful in documenting symptomatic arrhythmias, correlating them with clinical symptoms, detecting occult arrhythmias in asymptomatic patients with potentially lethal cardiac diseases, and monitoring changes with medical management in high-risk patients.

Recent technological advancements have led to a significant reduction in the size of these devices, making them more comfortable and less invasive for pediatric patients. The safety profile and efficacy of ILRs have earned them a place in the latest syncope and pediatric device guidelines [8]. However, ILRs have not been specifically tested for pediatric use, and there is scarce data on benefits in children and adolescence. This study seeks to bridge this knowledge gap by providing valuable insights into patient characteristics, indications for implantation, diagnostic yield, and subsequent medical management choices. Our findings are derived from a consecutive cohort of young patients who underwent Reveal LINQ II ILR implantation at a single pediatric center over a 7-year period.

- Hypothesis: The new generation of ILR contributes to significantly improve the diagnostic yield and management of undiagnosed cardiac symptoms in pediatric patients compared to conventional non-invasive methods.

## Materials and methods

This retrospective cohort study was conducted at a single pediatric cardiology center from January 2016 to December 2023. This study was conducted in accordance with the Declaration of Helsinki and the ethical standards of Bristol Royal Hospital for Children’s Institutional Review Board, including ethics approval. The study aimed to evaluate the effectiveness and safety of the Reveal LINQ II ILR in diagnosing and managing undiagnosed cardiac symptoms in pediatric patients. The study included patients under 18 years of age with unexplained symptoms such as palpitations, syncope, or seizure-like episodes, or those with a high risk of arrhythmias that had a Reveal LINQ II ILR implantation and had a follow-up of at least 12 months post-implantation. Most of these patients had undergone initial non-invasive monitoring, including a 24-h Holter monitoring, 7–10-day patch monitoring, and wearable rhythm monitoring (e.g., Kardia app or Apple Watch) without achieving a conclusive diagnosis, which were considered for ILR implantation. Exclusion criteria targeted patients who were lost to follow-up before 12 months post-implantation, as this would limit the comprehensive assessment of the ILR’s diagnostic yield and outcomes.

### Pre-implantation management

The pre-implantation cardiac screening approach was tailored to each case by the primary healthcare provider, following an individualized assessment. As a baseline, all patients underwent a minimum screening comprising a 12-lead ECG, a 24-h Holter monitoring, and echocardiography. For certain cases, additional investigations were conducted based on presenting symptoms, with options including an exercise stress test (EST), cardiac magnetic resonance imaging (CMRI), electrophysiology study (EPS), and/or electroencephalogram (EEG). The decision to pursue more extensive investigations was made on an individual basis.

### Indications for implantable loop recorder (ILR)

ILR indications encompassed a spectrum ranging from the diagnosis of infrequent or unexplained cardiac symptoms such as palpitations, chest pain, or seizure-like episodes to the follow-up of patients with previously recorded cardiac arrhythmias or those at high risk, such as those with ICCs.

### Essential features of ILR implantation technique

Reveal LINQ II ILRs (Medtronic Inc., Minneapolis, MN, USA) were implanted following standard sterile precautions in a dedicated cardiac catheterization laboratory. The procedures were performed under general or local anesthesia, depending on the patient’s age, cooperation, and preference. The implantation site was subcutaneous in the left parasternal region for all patients, except for one case where an axillary region implantation was chosen for aesthetic reasons. The devices were typically inserted at a 45-degree angle to the sternum, which is an approach chosen to enhance device stability and patient comfort. After sterile field preparation, the skin was infiltrated with local anesthetic, and the device was injected into the subcutaneous layer. The skin wound was closed with a resorbable single suture. Antibiotics were not routinely administered.

### Device programming and standardized follow-up

Following implantation, patient-specific programming of tachycardia and bradycardia detection zones was conducted, taking into consideration the patient’s age, underlying heart disease, medication, and clinical history. In selected patients with clinical evidence of non-sustained VT, the settings were adapted to enhance sensitivity and arrhythmia detection by reducing the rates to 180 bpm and the number of beats to 8 when necessary. Furthermore, patients and their families were trained to manually activate recordings during symptomatic episodes, allowing the capture of non-sustained VT that did not meet automatic detection criteria.

After recovering from anesthesia, patients and their family members were instructed on how to activate the monitor and transmit data, and were advised to contact the cardiology department in the event of symptoms. Patients were typically discharged on the same day following a 3- to 4-h observation period.

All patients performed a test download at home to ensure connectivity to the CareLink website. Test downloads were not included in the analysis. Transmissions were reviewed by dedicated pediatric cardiac rhythm physiologists, who forwarded them to pediatric electrophysiologists for evaluation in cases of potentially abnormal recordings.

The transmissions were subsequently subclassified into two subgroups: [1] diagnostic transmissions that reveal arrhythmias directly related to the primary reason for the ILR implantation and [2] relevant transmissions, including all significant findings, whether or not they are directly related to the primary reason for the ILR implantation. While such findings may not directly relate to the initial clinical suspicion, they still contribute valuable information to the overall clinical picture and patient management.

Elective follow-up was scheduled annually for patients with non-pathological transmissions, or more frequently as needed for those with positive or pathological findings.

For the purposes of this manuscript, relevant arrhythmias are defined as pauses of 3 s or longer, high-degree (type IIb or type III) atrioventricular (AV) block, and evidence of sinus node dysfunction (SND) based on age-related definitions, as well as supraventricular tachycardia (SVT), ventricular tachycardia (VT), or inappropriate sinus tachycardia (IST) [9, 10]. Non-relevant arrhythmias are defined as sinus bradycardia (SB), sinus pauses of less than 3 s, sinus tachycardia (ST), supraventricular ectopy (SVE), and ventricular ectopy (VE).

### Data collection

A retrospective chart review was conducted at our center following institutional review board approval. All patients under 18 years of age who underwent ILR implantation from January 2016 to December 2023 were included. Medical records were reviewed, and data were collected on patient demographics, family history, presence of congenital heart disease or genetic diagnosis, treatment, and ILR implantation indications. Device-related data collected included patient-activated and device-activated transmissions, recorded rhythm abnormalities, time from implantation to diagnostic transmission, patient management changes, and complications. For the purpose of this study, major complications were defined as those requiring significant medical intervention, prolonged hospitalization, or causing lasting harm; while minor complications included issues which could be resolved with minimal intervention.

### Statistical analysis

The results of this study are predominantly descriptive and are expressed as percentages, with mean values and standard deviations (SD) for continuous variables. A p-value of less than 0.05 was considered to indicate statistical significance. All statistical analyses were performed using the IBM SPSS Statistics 25.0 software (SPSS Inc., Chicago, IL, USA).

Differences between groups were evaluated using the chi-squared test and Fisher’s exact test for categorical variables. For continuous variables with a normal distribution, an unpaired Student’s *t*-test was used. For continuous variables without a normal distribution, the Mann–Whitney *U* test was applied. The Wilcoxon rank-sum test was employed for skewed continuous variables, and the ANOVA was used for comparisons among more than two groups.

## Results

**Fig. 1:**
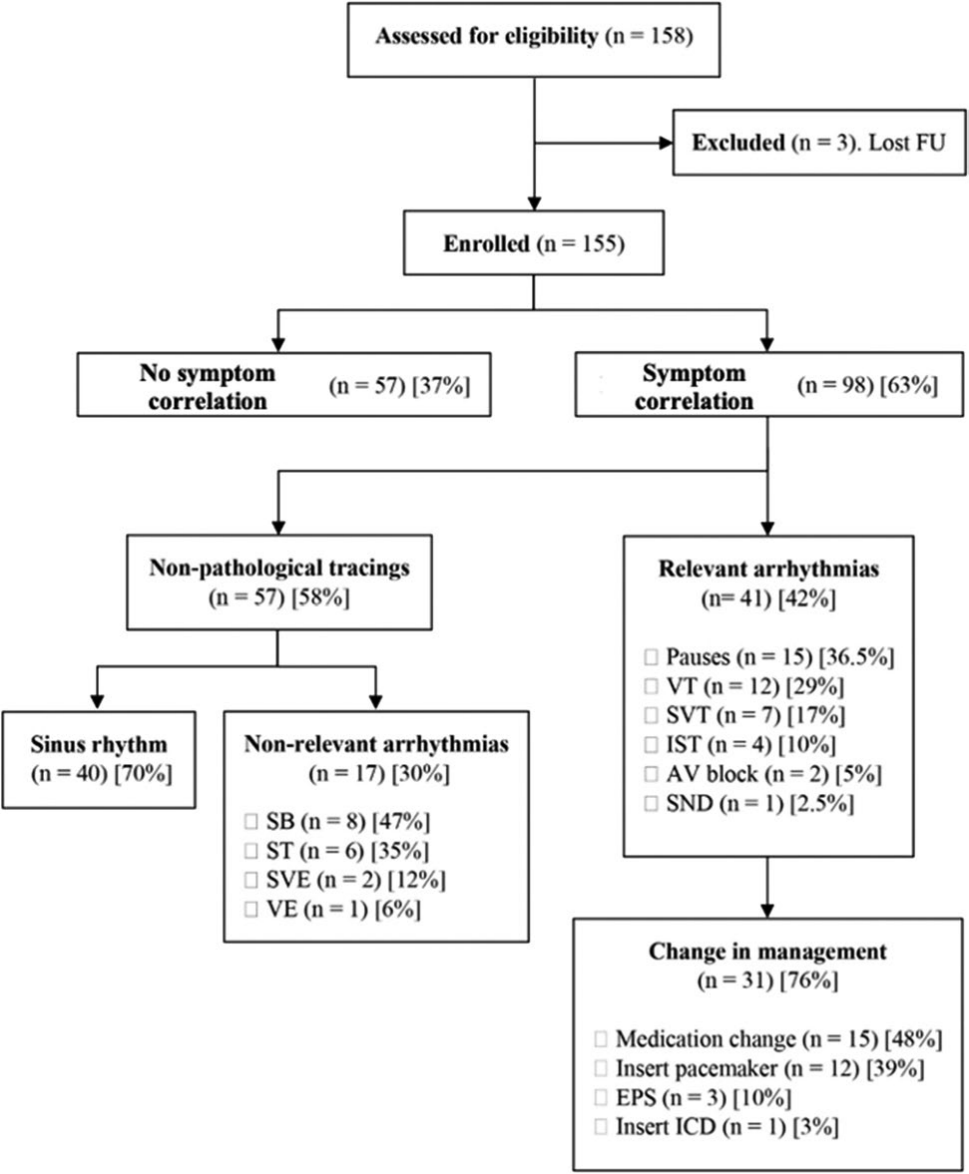
ILR outcomes flowchart. AV, atrioventricular; EPS, electrophysiology study; FU, follow-up; ICD, implantable cardioverter-defibrillator; ILR, implantable loop recorder; IST, inappropriate sinus tachycardia; SB, sinus bradycardia; SND, sinus node dysfunction; ST, sinus tachycardia; SVE, supraventricular ectopy; SVT, supraventricular tachycardia; VE, ventricular ectopy; VT, ventricular tachycardia

A total of 158 ILRs were implanted at a single center over a 7-year period. However, three patients were transferred to other institutions and subsequently lost to follow-up before 12 months post-implantation. This resulted in a final cohort of 155 patients for the study. Figure 1 shows a flowchart of the patients’ distribution and outcomes.

The baseline characteristics of the patients and the indications for implantation are detailed in Table 1.

**Table 1:** Demographic characteristics of patients with ILR implants

	**Total (*n* = 155)**	**Relevant arrhythmia (*n* = 41)**	**No/non-relevant arrhythmia (*n* = 114)**	***p* value**
Age (years)	10.3 ± 5.0	10.6 ± 5.1	10.3 ± 5.1	0.75
Sex (female)	74 (47.7%)	21 (51.2%)	53 (46.5%)	0.60
Weight (kg)	40.6 ± 21.6	42.8 ± 22.3	39.8 ± 21.3	0.44
Family history of SCA	18 (11.6%)	4 (10.0%)	14 (12.3%)	0.78
Family history	30 (19.4%)	3 (7.3%)	27 (23.7%)	**0.02**
ICC	21 (13.6%)	2 (4.9%)	19 (16.7%)	
CHD	3 (1.9%)	1 (2.4%)	2 (1.7%)	
Arrhythmia	3 (1.9%)	0 (0.0%)	3 (2.6%)	
Syndrome	3 (1.9%)	0 (0.0%)	3 (2.6%)	
Personal history	84 (54.2%)	21 (51.2%)	63 (55.3%)	0.65
ICC	31 (20.0%)	4 (10.0%)	27 (23.7%)	
CHD	18 (11.6%)	5 (12.2%)	13 (11.4%)	
Arrhythmia	28 (18.1%)	11 (26.8%)	17 (14.9%)	
Syndrome	12 (7.7%)	2 (4.9%)	10 (8.8%)	
Genetics	56 (36.1%)	13 (31.7%)	43 (37.7%)	0.49
Positive	43 (27.7%)	6 (14.6%)	37 (32.5%)	
Antiarrhythmic drugs	50 (32.5%)	15 (36.6%)	35 (30.7%)	0.49
Basal ECG (pathological)	33 (21.3%)	6 (14.6%)	27 (23.7%)	0.22
Holter (pathological)	20 (12.9%)	**7** (17.1%)	13 (11.4%)	0.35
EPS	25 (16.2%)	7 (17.1%)	18 (15.8%)	0.74
Ablation	15 (9.7%)	5 (12.2%)	10 (8.8%)	
Indication				0.17
Symptoms	100 (64.5%)	30 (75.0%)	70 (61.4%)	
Syncope	76 (49.0%)	24 (58.5%)	52 (45.6%)	
Palpitations	20 (12.9%)	6 (14.6%)	14 (12.3%)	
Seizure-like	4 (2.6%)	0 (0.0%)	4 (3.5%)	
High risk of arrhythmia	55 (35.5%)	11 (26.8%)	44 (38.6%)	
Known arrhythmia	35 (22.6%)	10 (24.4%)	25 (21.9%)	
ICC	12 (7.7%)	1 (2.5%)	11 (9.6%)	
Syndrome	5 (3.2%)	0 (0.0%)	5 (4.3%)	
SCA	3 (1.9%)	0 (0.0%)	3 (2.6%)	

*CHD*, congenital heart disease; *ECG*, electrocardiogram; *EPS*, electrophysiology study; *ICC*, inherited cardiac condition; *ILR*, implantable loop recorder; *SCA*, sudden cardiac arrest

The median age at implantation was 11.4 ± 5 years, ranging from 7 days to 17.9 years, with seven patients under the age of 1 year. Gender distribution showed no significant difference. Among the patients, half (*n* = 84) had a known baseline diagnosis, including an ICC, congenital heart disease, genetic syndrome, or a previously recorded cardiac arrhythmia. Table 2 details the specific distribution of the population as per the correlation of symptoms with tracings and the identification of relevant arrhythmias. Genetic testing conducted as part of their overall cardiac evaluation was performed in 56 patients (36%), with a positive yield in 43 cases (28% of the total population). The pathogenic/likely pathogenic genetic variants are outlined in Online Supplemental Table A.1. A comparison between subgroups of patients with relevant vs. non-relevant arrhythmias did not reveal any statistically significant differences in patients’ characteristics, treatment at implantation, or personal and/or family histories (Table 1).

**Table 2:** Personal history of patients with ILR implants (supplementary)

**ICC**	31 (20%)	CHD	18 (12%)	Arrhythmia	28 (18%)	Syndromes	12 (8%)
Long QT	18 (12%)	Complete AVSD	4 (2.6%)	VT	17 (11%)	Muscular dystrophy	3 (2.0%)
Brugada	6 (4.0%)	Tumor	3 (2.0%)	SVT	6 (2.6%)	Episodic ataxia syndrome	2 (1.3%)
DCM	2 (1.3%)	ASD	2 (1.3%)	AV block	3 (2.0%)	Tuberous sclerosis syndrome	2 (1.3%)
ARVC	2 (1.3%)	TGA	2 (1.3%)	SVT + VT	2 (1.3%)	Lennox syndrome	1 (1.3%)
CPVT	2 (1.3%)	Truncus	2 (1.3%)			Proteus syndrome	1 (1.3%)
Short QT	1 (0.7%)	PS	1 (0.7%)			Gorlin syndrome	1 (1.3%)
		AS	1 (0.7%)			Dravet syndrome	1 (1.3%)
		ToF	1 (0.7%)			Ehlers-Danlos syndrome	1 (1.3%)
		Ebstein anomaly	1 (0.7%)				
		CoA with AAH and VSD	1 (0.7%)				
**No relevant personal history 66 (42%)**							

*AHH*, aortic arc hypoplasia; *ARVC*, arrhythmogenic right ventricle cardiomyopathy; *AS*, aortic stenosis; *ASD*, atrial septal defect; *AVSD*, atrioventricular septal defect; *CoA*, coarctations of the aorta; *CPVT*, catecholaminergic polymorphic ventricular tachycardia; *DCM*, dilated cardiomyopathy; *ICC*, inherited cardiac condition; *PS*, pulmonary stenosis; *SVT*, supraventricular tachycardia; *TGA*, transposition of the great arteries; *ToF*, tetralogy of Fallot; *VSD*, ventricular septal defect; and *VT*, ventricular tachycardia

Regarding the type of transmissions performed with the device, all patients conducted an initial transmission within 3 days of device implantation. During the follow-up period, 98 patients (63% of the total cohort) activated recordings during symptoms, leading to 41 (42%) of these transmissions revealing diagnostic arrhythmia recordings. Conversely, device-activated transmissions occurred in 33 patients (21% of the total cohort) and showed relevant arrhythmia tracings in 26 (79%). Among the patients without a diagnosed arrhythmia (*n* = 57), 70% of the transmissions exhibited normal sinus rhythm, while the remaining recordings showed minor findings such as sinus bradycardia, sinus tachycardia, and isolated supraventricular or ventricular ectopy. Table 3 presents the distribution of patients by type of device transmissions in a graphical format.

**Table 3:** Classification based on devise transmissions

Type of transmission	Number (percentage of total cohort)	
Patient-activated transmissions	Initial transmission	155 (100%)
	Follow-up transmission	98 (63%)
Device-activated transmissions	33 (21%)	
No transmissions during follow-up	24 (15%)	

The median time from device implantation to a diagnostic transmission was 175 days, with a total median follow-up of 845 days (2.3 years). Among patients with relevant arrhythmias, the most common abnormality was sinus pauses (*n* = 15, 37.5%), with a length ranging from 4 to 30 s (10.7 ± 7.3 s), and 73% being both patient- and device-activated. The second most frequent arrhythmia was ventricular tachycardia (VT) (*n* = 12, 30%), of which 83% were non-sustained VT.

**Fig. 2:**
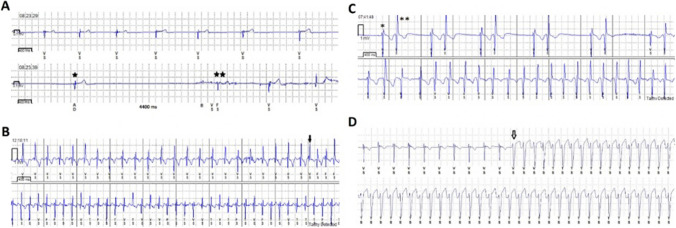
Example ILR tracings demonstrating diagnostic recordings. **A** 9-year-old patient with long QT syndrome presenting with repetitive dizziness and syncope. Note the profound bradycardia at the beginning of the tracing followed by a 4.4-s pause (delimitated by the stars). **B** 14-year-old patient presenting with occasional palpitations. Note that the initial rhythm is sinus tachycardia at a rate of 150 bpm, followed by supraventricular tachycardia at 250 bpm, triggered by a premature atrial contraction (black arrow). **C** This tracing belongs to a 12-year-old patient with left ventricular non-compaction who had presented with non-sustained ventricular tachycardia (VT) on 24-h Holter monitoring. Note the initial bigeminy, which consists of a sinus beat (asterisk) followed by a ventricular premature beat (double asterisk), followed by VT at the end of the tracing. Even though the QRS morphology is not significantly wide, it differs from the QRS in sinus rhythm and matches the QRS morphology identified as isolated ventricular premature beats in the upper portion of the recording. These observations collectively support a diagnosis of ventricular origin for the rapid rhythm in the lower portion of the tracing. **D** 5-month-old patient presenting with idiopathic fascicular ventricular tachycardia. Note the presence of normal sinus rhythm at a rate of 130 bpm at the beginning of the tracing, followed by a monomorphic ventricular tachycardia at a rate of 225 bpm (white arrow)

In the subgroup consisting of 76 patients presenting with syncope, 23 (30%) experienced relevant arrhythmias, including 14 cases of significant pauses, 4 cases of VT, 2 cases of SVT, 2 cases of IST, and 1 case of AV block. Among the 20 patients presenting with palpitations, 7 (35%) exhibited relevant arrhythmias, consisting of 3 cases of SVT, 2 cases of IST, 1 case of VT, and 1 case of significant pause. In the high-risk group of 12 patients with ICC, the monitor identified one episode of polymorphic ventricular arrhythmia in 1 patient. Figure 2 provides electrocardiographic tracing samples from the most relevant ILR transmissions in the patient cohort.

A total of 47 patients (30% of total cohort) diagnosed through ILR monitoring benefited from targeted arrhythmia management, which included medication adjustments, device implantation, and EPS.

Specifically, the arrhythmias identified in the ILR tracings led to changes in management for several patients: 15 patients (10%) had their medication adjusted, primarily with antiarrhythmic drugs to treat detected tachyarrhythmias; 12 patients (8%) received pacemaker implants due to symptomatic bradycardia or pauses; 3 patients (2%) were referred for an electrophysiology study (EPS); and 1 patient (1%) was indicated for an implantable cardioverter defibrillator (ICD) (Table 2).

In the group that received medical treatment, the adjustments primarily involved antiarrhythmic medications aimed at controlling tachyarrhythmias identified in the ILR tracings. The 12 patients who received pacemakers did so based on criteria related to symptomatic bradycardia or pauses detected through ILR transmissions, highlighting the diagnostic value of the device in guiding these treatment decisions. The three patients who underwent EPS were initially diagnosed with syncope or palpitations of unknown etiology. The ILR tracings identified supraventricular arrhythmias, enabling targeted treatment with percutaneous ablation. The patient who received an ICD was a high-risk individual with CPVT, where ILR transmissions revealed non-sustained polymorphic ventricular arrhythmia despite optimal medical therapy, warranting the intervention.

**Fig. 3:**
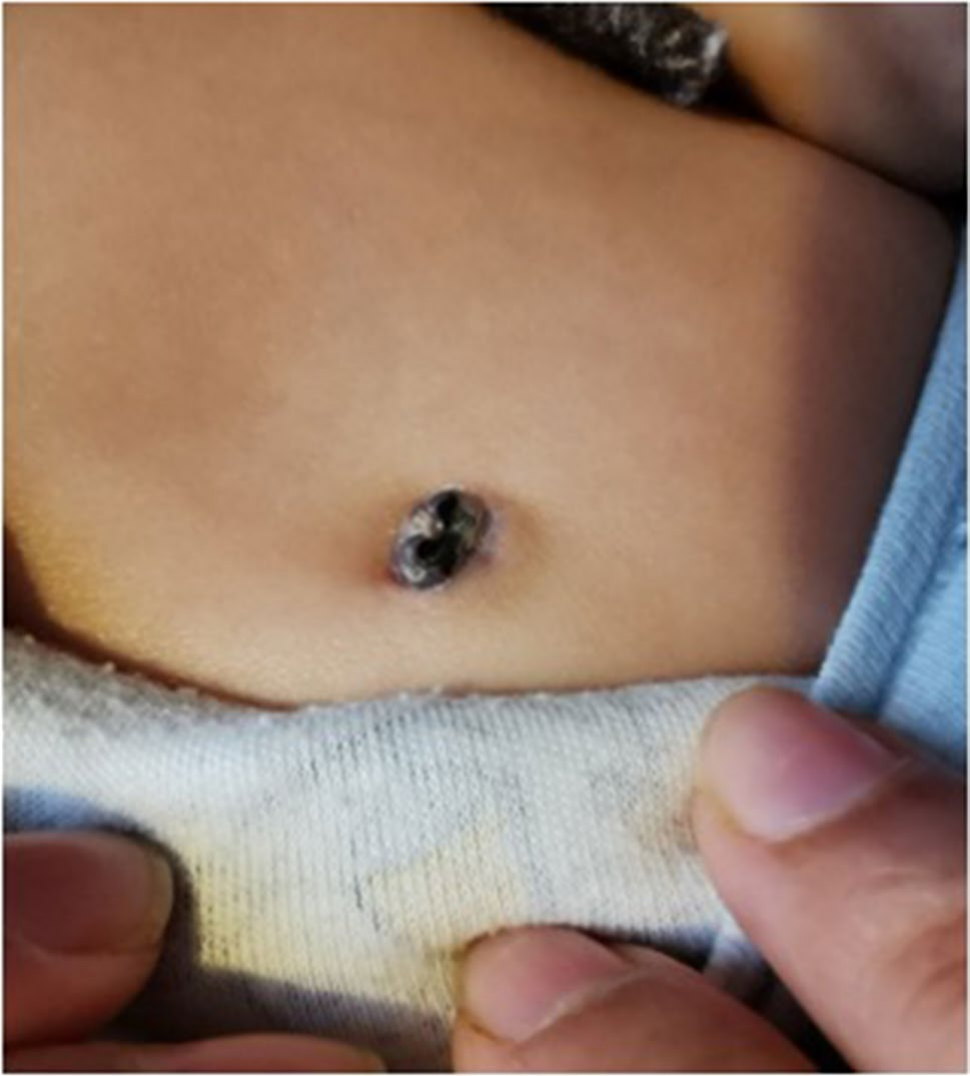
Skin erosion and device explant in a 4-month-old boy after 2 months of the initial implantation

Throughout the device implantation and follow-up duration, no significant complications were recorded. Minor complications were observed in four patients (2.5%). These included skin erosion in a 4-month-old infant (Fig. 3), device dysfunction, and an allergic reaction, necessitating device removal. It is important to note that one patient passed away during the follow-up period, but this was unrelated to cardiac symptoms or arrhythmia. The skin erosion in the 4-month-old patient was classified as a minor complication due to its early identification and prompt management, which prevented prolonged hospitalization or lasting harm. The device was removed and replaced under sedation, avoiding the need for general anesthesia.

## Discussion

The present study sheds light on the role of ILRs in elucidating arrhythmias and guiding management in the pediatric patients. Arrhythmia-related symptoms, such as palpitations, chest pain, and syncope, are often difficult to interpret and diagnose in this population. The diverse range of ILR indications reflects the need to address various clinical scenarios and underscores the importance of personalized and targeted monitoring strategies. Traditional diagnostic tools, including ECGs and Holter monitors, often yield around 5% diagnostic success due to the brief and self-limited nature of the episodes [11, 12]. The integration of ILRs, external loop recorders (ELRs), and remote telemetry in a novel technology strategy has significantly increased the diagnostic yield to 50% and 80% at 2 and 4 years, respectively [13].

The 2021 Pediatric and Congenital Electrophysiology Society (PACES) Expert Consensus Statement on the indications and management of cardiovascular implantable electronic devices in pediatric patients underscores the growing importance of ILR in pediatric cardiology and electrophysiology [8]. Class I recommendations advocate for ILR implantation in high-risk patients with syncope, while Class IIa recommendations include cases of recurrent syncope of uncertain origin, infrequent symptoms suspected to be due to an arrhythmia, or for guiding the management of patients with ICC (also referred to as channelopathies), or structural heart diseases.

Advancements in ILR technology, such as smaller size, lighter weight, MRI compatibility, and extended longevity, have significantly enhanced diagnostic capabilities [14]. Our study aligns with previous data, affirming ILRs as a strategic tool for guiding management and offering reassurance to patients and physicians [15–17]. Moreover, the options of remote programming and mobile applications are particularly helpful for teenagers.

Our study demonstrated a symptom-to-tracing correlation in 63% of the implanted patients, achieving a diagnostic yield for arrhythmias in 60% of this cohort. Notably, 30% of these patients benefited from an ILR-related arrhythmia diagnosis, which led to the implementation of targeted management plans, including medication adjustments, device implantation, and EPS. Additionally, ILRs played a crucial role in monitoring patients who had experienced severe forms of syncope, facilitating the diagnosis of severe breath-holding spells, seizures, and potentially lethal arrhythmias, which indicated the need for an implantable cardioverter-defibrillator (ICD).

New ILR algorithms exhibit remarkable efficacy in detecting abnormal heart rhythms in children, as evidenced by an 80% positive diagnostic yield in device-activated transmissions. Moreover, in patients without arrhythmia identification, excluding abnormal rhythms during symptoms has a positive psychological impact, providing reassurance to patients and their families. Further investigation is warranted to assess the impact of heart monitoring on the quality of life using ILRs.

In our study, the subgroup of infants (< 1 year) presented unique challenges related to the implantation and monitoring process. One notable complication was the occurrence of a wound opening and extrusion of the device in a 4-month-old infant. The small thoracic volume and thinner subcutaneous tissue in this age group can increase the risk of such local wound complications. However, these issues are largely preventable with careful attention to proper implant techniques and meticulous skin closure.

The extended monitoring period provided by ILRs is essential for accurately diagnosing and managing sporadic arrhythmic events, as evidenced by the median time of 175 days from implantation to diagnostic tracing. This highlights the importance of more flexible insurance approval processes in some regions, ensuring they align with clinical guidelines and meet patient needs effectively.

Finally, our experience is in accordance with previous research [18–22], proving the safety of the implantation procedure with a low rate of complications.

## Limitations

This study is limited by its retrospective design, analysis of only one ILR vendor, the variety of ILR indications and patient characteristics, and the ongoing follow-up of some patients who may develop arrhythmic events at any moment**.**

## Conclusions

Implantable loop recorders (ILRs) have proven to be both effective and safe in the diagnosis and management of pediatric arrhythmias. The latest advancements in device miniaturization and new arrhythmia detection algorithms offer significant benefits to the pediatric population. Even when a pathological ECG tracing is not demonstrated, ILRs provide reassurance to patients, families, and physicians. Further studies are needed to assess the impact of ILRs on the quality of life and sport participation in high-risk young patients.

## Supplementary Information

Below is the link to the electronic supplementary material.

Supplementary file1 (PDF 95 KB)

## Data Availability

No datasets were generated or analysed during the current study.

## Abbreviations

CMRI: Cardiac magnetic resonance imaging
ECG: Electrocardiogram
EEG: Electroencephalogram
ELR: External loop recorder
EPS: Electrophysiology study
EST: Exercise stress test
ICC: Inherited cardiac conditions
ICM: Implantable cardiac monitors
ILR: Implantable loop recorder
