# Supplementary material for: Role of new generation implantable loop recorders in managing undiagnosed pediatric cardiac symptoms
Source: Eur J Pediatr. 2024 Aug 19;183(10):4553–61. doi: 10.1007/s00431-024-05728-8 (PMC11413195; doi:10.1007/s00431-024-05728-8)
Supplement: Supplementary file 1 — Supplementary file1 (PDF 95 KB) [file 431_2024_5728_MOESM1_ESM.pdf]

Supplementary Material

Table A.1. Genetic background (n = 43)

Variant	N	Clinical syndrome
SCN5A	9 (6.0%)	Brugada syndrome and long QT type 3
CACNA1C	3 (2.0%)	Long QT type 8 (Timothy syndrome)
KCNH2	3 (2.0%)	Long QT type 2
KCNQ1	3 (2.0%)	Long QT type 1 and CPVT
KCNJ2	3 (2.0%)	Long QT type 7 (Andersen-Tawil syndrome)
SCN1B	2 (1.3%)	Brugada syndrome
CACNA1A	2 (1.3%)	Neurological disorders and cardiac arrhythmias
DSP	2 (1.3%)	ARVC
RYR2	2 (1.3%)	ARVC, DCM and CPVT
TSC2	2 (1.3%)	Tuberous sclerosis syndrome
CALM2	1 (0.7%)	Long QT type 15
Del 2q37	1 (0.7%)	Neurological disorder
DMPK	1 (0.7%)	Steinert myotonic dystrophy
FLNA	1 (0.7%)	Ehlers-Danlos syndrome
FXN	1 (0.7%)	Friedreich ataxia
HERG	1 (0.7%)	Long QT type 2
LMNA	1 (0.7%)	Congenital muscular dystrophy
NKX2.5	1 (0.7%)	CHD and AV block
PTCH1	1 (0.7%)	Gorlin syndrome
RYR1	1 (0.7%)	Congenital myopathy and arrhythmias
SCN1A	1 (0.7%)	Dravet syndrome
SLC22A5	1 (0.7%)	Primary carnitine deficiency, cardiomyopathy and short QT

ARVC = arrhythmogenic right ventricle cardiomyopathy; CHD = congenital heart disease; CPVT = catecholaminergic polymorphic ventricular tachycardia; DCM = dilated cardiomyopathy.
